# Development of Federally Mandated Risk Evaluation and Mitigation Strategies (REMS) for Transmucosal Immediate-Release Fentanyl Products

**DOI:** 10.1111/papr.12040

**Published:** 2013-03-06

**Authors:** Joseph V Pergolizzi, Christopher G Gharibo, Jeffrey A Gudin, Srinivas R Nalamachu

**Affiliations:** * Department of Pharmacology, Temple University School of MedicinePhiladelphia, Pennsylvania, U.S.A; † Department of Medicine, Johns Hopkins University School of MedicineBaltimore, Maryland, U.S.A; ‡ Department of Anesthesiology, Georgetown University School of MedicineWashington, District of Columbia, U.S.A; § Naples Anesthesia and Pain AssociatesNaples, Florida, U.S.A; ¶ Departments of Anesthesiology and Orthopedics, Pain Medicine, NYU Langone-Hospital for Joint DiseasesNew York, New York, U.S.A; ** Pain Management and Palliative Care at Englewood Hospital and Medical CenterEnglewood, New Jersey, U.S.A; †† International Clinical Research InstituteOverland Park, Kansas, U.S.A

The potential adverse health consequences of prescription opioid analgesics are well documented and include abuse, addiction, and death due to overdose.^1^–^3^ In 2007, the US Congress passed the Food and Drug Administration Amendments Act (FDAAA), which authorized the Food and Drug Administration (FDA) to require that drug manufacturers implement a Risk Evaluation and Mitigation Strategy (REMS) for pharmaceuticals with known or suspected risks of abuse and overdose, although REMS were not required for short-acting opioids (eg, combination oxycodone/acetaminophen).^4^–^7^ FDAAA required that the REMS for each drug be designed with the purpose of evaluating and mitigating the risks of adverse events. This editorial briefly describes the development of a shared REMS for transmucosal immediate-release fentanyl (TIRF) products from individual REMS (with examples from a previous, separate program for sublingual TIRF tablets [Abstral®]), implementation of the shared REMS, and remaining challenges.

The goals of the TIRF REMS, which is required by the FDA, are to ensure patient access to important medications and to “mitigate the risk of misuse, abuse, addiction, overdose and serious complications due to medication errors.”[Bibr b8] In 2009, the FDA released a draft “Guidance for Industry” describing the requirements for how a REMS should attempt to achieve these goals.^9^ Although problems with opioid analgesics range from accidental medical misuse (eg, through treatment noncompliance) to intentional illegal use, the new REMS policies for opioids focus primarily on safe and appropriate medical use. The FDAAA and the 2009 Guidance recommended “Elements to Assure Safe Use” (ETASU); this document refers to a variety of methods to promote safe medication access.[Bibr b6] However, a REMS program that imposes undue restrictions on appropriate opioid treatment could negatively affect the adequate care of all patients who may need these medications.^10^ The law therefore also obligates the FDA to ensure, via periodic evaluations, that REMS requirements do not limit medication access or create an undue burden on the healthcare system.[Bibr b6]

A separate REMS for sublingual TIRF tablets, which received final approval from the US FDA in January 2011,[Bibr b11] served as one model for the shared REMS that has now been implemented for all TIRF products. These sublingual fentanyl tablets are a Schedule II controlled substance with an abuse liability similar to that of other legal or illicit opioid agonists.^12^ They are approved only for the management of breakthrough pain in patients, 18 years of age and older, who are already receiving and are tolerant to opioid therapy for underlying persistent cancer pain. The stated goals of the REMS for the sublingual TIRF tablets, identical to those of the current shared TIRF REMS Access program (hereafter referred to as TIRF REMS), were to “mitigate the risk of misuse, abuse, addiction, overdose and serious complications due to medication errors.”[Bibr b13] As with the shared TIRF REMS, the goals of the sublingual TIRF tablets REMS were to be achieved by the following actions:

1. Prescribing and dispensing only to appropriate patients, which includes use only in patients with cancer who are opioid tolerant.
2. Preventing inappropriate conversion between fentanyl products.
3. Preventing accidental exposure to children and others for whom it was not prescribed (ie, reducing the incidence of adverse events associated with accidental exposures and use by nontolerant individuals).
4. Educating prescribers, pharmacists, and patients on the potential for misuse, abuse, addiction, and overdose.

Although an ETASU plan can suggest various methods to address these issues, the available strategies are open to some criticism. For example, a REMS program may limit opioid distribution to certain healthcare settings, specially certified registrant pharmacies, or patients listed in a registry.^14^ However, although opioid-associated misuse, abuse, addiction, and mortality have increased contemporaneously with prescribing, the exact relationship between medical and nonmedical prescription opioid use remains unclear.^15^ Training can be required, but there is limited evidence for the efficacy of educational efforts. The components of the TIRF REMS that attempt to modify illegal behavior by patients represent an extension of FDA authority that has not been seen previously. The complexity of nonmedical use of prescription opioids, coupled with the unproven and unknown effects of a REMS, have led to skepticism that there is sufficient understanding of how to develop an effective and safe REMS.^5^ Although the FDA is mandating these mitigation strategies now in an attempt to reduce adverse events related to the use of prescription opioids, close attention to their intended and unintended consequences is warranted in the future.

## Implementation of the Shared TIRF REMS Program

A single, uniform, shared REMS was developed by manufacturers of TIRF products, as required by the FDA.[Bibr b16] Under the shared TIRF REMS, outpatient utilization of these medications is controlled by a limited-access distribution system that mandates enrollment and certification of prescribers, pharmacies, distributors, and patients. Additionally, inpatient ordering and dispensing require pharmacy certification. The shared TIRF REMS was approved by the FDA on December 28, 2011,[Bibr b16] and implementation began on March 12, 2012.[Bibr b17] Pharmacies and prescribers were automatically transferred from the previous REMS to the current shared TIRF REMS. Clinicians who prescribe TIRF products to outpatients must meet several ETASU certification requirements,[Bibr b6] which include reviewing educational materials, successfully completing a *Prescriber Knowledge Assessment*, and completing and signing a *Prescriber Enrollment Form*. However, for inpatient use, the patients and prescribers are not required to be enrolled, although the pharmacy must register with the program.

An important objective for the shared TIRF REMS is to reduce the incidence of adverse events associated with accidental exposure and use by nontolerant individuals. Accordingly, a program has been designed to educate prescribers about appropriate indications, dosing and administration, conversion between TIRF products, and patient selection, as well as the risk of and behaviors associated with abuse, addiction, and diversion. A single set of Educational Program materials and Knowledge Assessment forms has been developed for the entire TIRF drug class.[Bibr b8] Prescribers are offered an applied learning opportunity in which topics are introduced via patient vignettes designed to elicit treatment decisions based on presented clinical information. Prescribers must repeat the enrollment requirements at least once every 2 years to maintain their certification. However, the actual impact of the REMS educational program in assuring acceptable prescribing, enhanced medication security, and avoidance of accidental exposures is not known because no validation testing is currently available. As required by the TIRF REMS program, a survey has been sent to prescribers; results of this survey will be collected until October 30, 2012, and may begin to answer some of these questions.

All patients (excluding inpatients) and their healthcare providers must complete and sign a *Patient-Prescriber Agreement Form* (PPAF), which must be sent to the TIRF REMS program within 10 working days from when the patient's first prescription is processed. Within these 10 days, a maximum of three prescriptions are allowed until the completed PPAF is received. The PPAF contains numerous statements for attestation designed to reduce adverse events resulting from inappropriate use. This PPAF differs from typical opioid agreements in that it is used as an educational tool that is designed to facilitate conversation between the patient and prescriber; a key aspect is that prescribers are required to counsel each patient and provide a Medication Guide relating to the prescribed product. It is required that patients and providers sign the agreement to receive or prescribe the drug, respectively. A new PPAF must be completed at least once every 2 years.

All ETASU requirements discussed thus far have related solely to prescribers and their outpatients, but it is important to note that enrollment and certification are mandatory for all pharmacies that intend to distribute TIRF products to outpatients.[Bibr b18] Enrollment conditions include designating an authorized pharmacist to be responsible for training relevant staff. For prescriptions issued on an outpatient basis, a pharmacy management system (PMS) must be enabled to support communication with the REMS system. Any pharmacist in a certified pharmacy who has undergone the relevant training can process TIRF prescriptions through the PMS. Confirmation of patient, pharmacy, and physician enrollment is provided through electronic verification.[Bibr b18] Each patient receiving an initial TIRF prescription is passively enrolled via the PMS and may procure a prescription from any pharmacy enrolled in the TIRF REMS program. The enrollment activities summarized here are the essential elements of the approved TIRF REMS program for outpatient prescribing. As stated earlier, for inpatient use only, the pharmacy must register with the program, but patients and prescribers are not required to be enrolled.

## Existing Challenges and Value Positioning

The foundation of the REMS program is to address public health issues with prescription opioids by focusing principally on prescribing and patient education. However, even a REMS constructed for the entire class of prescription opioids,^14^ rather than for groups of opioids or individual products, should only be considered an incomplete response to the misuse and abuse of these drugs. The success of the current shared TIRF REMS program may be limited because it does not address alternate procurement methods that sidestep prescriber–patient contact.^19^–^21^ More data on the relative magnitude of medical and nonmedical aspects of abuse and misuse are needed, as well as better understanding of the populations that divert and abuse opioids.^22^ At this time, additional information of this type is limited, as effectively illustrated in an analysis of public commentary submitted to the FDA through an open docket relating to a shared REMS for long-acting and extended-release opioid products.^23^ Even with better understanding of illicit opioid use, developing effective abuse and diversion minimization systems would likely be difficult.

The 2009 report from the US Drug Enforcement Administration (DEA) National Drug Intelligence Center detailed multiple sources of diversion, such as robberies during transport, thefts (including those by healthcare workers) from pharmacies and healthcare facilities, and “rogue” Internet pharmacies.^24^ Furthermore, the security of medications dispensed to patients remains an important issue; the National Survey of Drug Use and Health demonstrated that most people (71%) reporting nonmedical opioid use received the drug from a friend or family member, making it difficult to conclude whether a prescriber or patient was at fault.^25^ In sum, activities that do not involve prescribers directly (eg, misuse, abuse, intentional and unintentional diversion) nevertheless can contribute to adverse consequences associated with opioid medications. Recognition of these contributing factors has prompted the federal government to issue a *National Drug Control Strategy* aimed at promoting the safe disposal of unused or unneeded pharmaceuticals.^26^ Such a policy corresponds to the DEA's supervision of the first national take-back program to reduce residual stores of controlled drugs in homes,^27^ an initiative that has been repeated 4 times, most recently in September 2012.[Bibr b28] Although it is hoped that these approaches for identifying and responding to the multitude of potential diversion sources cumulatively represent a critical advantage over previous reaction to the phenomenon,^29^ few outcomes data are yet available.

The question of how to define and assess the “success” of any REMS program remains a difficult one. A strongly restrictive REMS might reduce deleterious opioid-related consequences, such as overdose; however, it could be challenging to determine whether this benefit comes largely from successfully impeding nonmedical use or from drastically limiting appropriate medical availability, which might be unacceptable. Situations such as these, in light of the FDA's legal obligation to authorize REMS programs that adequately manage the risks of medications without unduly burdening the healthcare system or reducing patient access,[Bibr b6] will continue to generate debate, especially if uniform approaches are not implemented for similar products.

## Conclusion

The FDA's efforts to devise a strategy aimed at mitigating deleterious consequences from the inappropriate or nonmedical uses of TIRF products and other prescription opioids are commendable. Unfortunately, there is an incomplete understanding about how legitimate prescribing practices contribute to nonmedical analgesic use. The goal of reducing abuse, misuse, addiction, and overdose deaths, balanced against the potential for exacerbating untreated or poorly treated pain, must remain a central imperative to the process of REMS development, as the FDA itself has recognized.^14^ The complexity of this goal is such that it cannot be achieved in isolation. A multiagency intervention seems prudent and justified, especially given the national public concern related to prescription opioid nonmedical use and diversion. Collaboration among the key stakeholders, including the FDA, the DEA, the Centers for Disease Control and Prevention, the Substance Abuse and Mental Health Services Administration, the Federation of State Medical Boards, individual state and local healthcare regulatory and law enforcement agencies, prescribers, pharmacies, patient advocacy groups, and product manufacturers, has the potential to improve appropriate prescribing of opioids while helping to mitigate the risks of abuse and misuse. The literature supports the need for a comprehensive solution to the complex problems of nonmedical use and overdose with opioids.^15^ The new shared TIRF REMS program may be an important first step toward this goal, but it is likely that additional measures will be needed in the future.
